# Supplementary material for: Exploring the CNOT1(800–999) HEAT Domain and Its Interactions with Tristetraprolin (TTP) as Revealed by Hydrogen/Deuterium Exchange Mass Spectrometry
Source: Biomolecules. 2025 Mar 11;15(3):403. doi: 10.3390/biom15030403 (PMC11939966; doi:10.3390/biom15030403)
Supplement: Supplementary file 1 [file biomolecules-15-00403-s001.zip › biomolecules-3488387-supplementary.pdf]

Supplementary Materials

Exploring the CNOT1(800-999) HEAT domain and its interactions with tristetraprolin (TTP) as revealed by hydrogen/deuterium exchange mass spectrometry

Maja K. Cieplak-Rotowska,[1,2] Michał Dadlez,[3] and Anna Niedzwiecka[2,]*

[1] *Division of Biophysics, Institute of Experimental Physics, Faculty of Physics, University of Warsaw, 02-089 Warsaw, Poland*

[2] *Laboratory of Biological Physics, Institute of Physics, Polish Academy of Sciences, Aleja Lotnikow 32/46, PL-02668 Warsaw, Poland*

[3] *Laboratory of Mass Spectrometry, Institute of Biochemistry and Biophysics, Polish Academy of Sciences, PL-02106 Warsaw, Poland*

* annan@ifpan.edu.pl (A. Niedzwiecka)

Table S1. The primers used for generation of the E893A/Y900A and E893Q/Y900H point mutants of CNOT1(800-999).

	**Forward**	**Reverse**
**E893A**	AAGGAACTTGTTTGAAGCATATCGTTTTTTTCCC	GGGAAAAAAACGATATGCTTCAAACAAGTTCCTT
**E893Q**	AAGGAACTTGTTTGAGCAATATCGTTTTTTTCCC	GGGAAAAAAACGATATTGCTCAAACAAGTTCCTT
**Y900A**	TCGTTTTTTTCCCCAGGCTCCTGATAAAGAGTT	AACTCTTTATCAGGAGCCTGGGGAAAAAAACGA
**Y900H**	TCGTTTTTTTCCCCAGCATCCTGATAAAGAGTT	AACTCTTTATCAGGATGCTGGGGAAAAAAACGA

Table S2.

Kinetic parameters of hydrogen-deuterium exchange measured for CNOT1(800-999) at 2 µM; ND_i, maximal number of hydrogens exchanging at the corresponding HDX rate constant, k_i; *ND*, maximum number of hydrogens exchanged; $D_{\%}$, percent of the exchangeable amide hydrogens that undergo exchange to deuterium; *AIC %,* probability (in %) that the selected model is correct based on the Akaike's Information Criteria; δ, one standard deviation resulting from numerical fit. Data corrected for backexchange.

		Best-fit values																
Res. no.	Sequence	ND_1 [Da]	δND_1	k_1 [min^{-1}]	δk_1	ND_2 [Da]	δND_2	k_2 [min^{-1}]	δk_2	ND_3 [Da]	δND_3	k_3 [min^{-1}]	δk_3	ND [Da]	δND	$D_{\%}$	δ $D_{\%}$	% AIC
800-818	NNDPFVQRKLGTSGLNQPT									17.42	0.05	24	2	17.42	0.05	108.9	0.3	86
800-819	NNDPFVQRKLGTSGLNQPTF									19.18	0.12	27	7	19.18	0.12	112.8	0.7	86
800-820	NNDPFVQRKLGTSGLNQPTFQ									19.71	0.07	27	4	19.71	0.07	109.5	0.4	86
800-824	NNDPFVQRKLGTSGLNQPTFQQTDL									24.76	0.09	29	5	24.76	0.09	112.5	0.4	86
820-824	QQTDL									4.380	0.012	20.8	1.2	4.380	0.012	109.5	0.3	85
825-835	SQVWPEANQHF									9.79	0.04	18.7	1.1	9.79	0.04	108.9	0.4	> 99
825-838	SQVWPEANQHFSKE									13.82	0.04	15.5	0.5	13.82	0.04	115.2	0.4	> 99
825-847	SQVWPEANQHFSKEIDDEANSYF	8.5	0.4	0.043	0.005					12.1	0.3	12.4	1.5	20.7	0.2	98.3	1.0	> 99
828-838	WPEANQHFSKE									10.56	0.09	18	2	10.56	0.09	117.3	1.0	98
829-838	PEANQHFSKE									10.56	0.09	16.5	1.7	10.56	0.09	117.3	1.0	99
839-846	IDDEANSY	4.5	0.2	0.041	0.005	2.74	0.18	5.2	1.0					7.22	0.11	103.1	1.6	> 99
839-847	IDDEANSYF	5.1	0.3	0.040	0.005	3.0	0.2	5.2	1.1					8.04	0.14	100.5	1.8	> 99
846-859	YFQRIYNHPPHPTM	5.8	0.3	0.047	0.007	4.0	0.3	8.3	2.0					9.84	0.19	98.4	1.9	> 99
847-859	FQRIYNHPPHPTM	4.75	0.19	0.056	0.006	4.44	0.17	6.4	0.7					9.19	0.10	102.1	1.2	> 99
847-861	FQRIYNHPPHPTMSV	4.5	0.2	0.053	0.007	6.59	0.19	7.1	0.6					11.07	0.12	100.6	1.1	> 99
860-865	SVDEVL	1.16	0.17	0.08	0.03	1.34	0.15	7	2					2.51	0.09	50.1	1.8	99
862-867	DEVLEM	HDX n.d.														0		
863-867	EVLEM	HDX n.d.														0		
864-868	VLEML	HDX n.d.														0		
866-883	EMLQRFKDSTIKREREVF	3.5	0.2	0.0098	0.0018					3.93	0.13	11.4	1.9	7.4	0.2	43.7	1.4	> 99
868-883	LQRFKDSTIKREREVF	3.02	0.20	0.024	0.006					4.12	0.17	18	7	7.14	0.15	47.6	1.0	> 99
868-886	LQRFKDSTIKREREVFNCM	3.4	0.3	0.038	0.010					3.91	0.20	30[a]		7.31	0.20	40.6	1.1	90
869-883	QRFKDSTIKREREVF	2.79	0.15	0.025	0.005					4.53	0.13	14	2	7.32	0.11	52.3	0.8	> 99
886-893	MLRNLFEE	3	3	0.0012	0.0015									3	3	50	50	> 99
887-893	LRNLFEE	1.7	0.2	0.0034	0.0007									1.7	0.2	28.8	3.8	> 99
887-894	LRNLFEEY	2.8	1.0	0.0029	0.0016									2.8	1.0	40	15	> 99
888-893	RNLFEE	0.32	0.16	0.1[a]						0.19	0.12	30[a]		0.51	0.10	10.1	1.9	86
894-904	YRFFPQYPDKE	5.8	0.3	0.015	0.003	3.3	0.2	6.1	1.4					9.0	0.3	100	3	> 99
894-905	YRFFPQYPDKEL	5.4	0.6	0.008	0.003	4.8	0.5	1.1	0.3					10.2	0.6	102	6	> 99
894-909	YRFFPQYPDKELHITA	8.1	0.4	0.032	0.005	3.2	0.4	5.1	1.8					11.2	0.3	80.1	1.8	> 99
912-917	FGGIIE	HDX n.d.														0		
912-919	FGGIIEKG	2.44	0.17	0.0075	0.0012					0.18	0.06	30[a]		2.63	0.17	38	2	89
912-922	FGGIIEKGLVT	4.47	0.13	0.0170	0.0016	1.23	0.10	6.4	1.6					5.70	0.11	57.0	1.1	> 99
912-924	FGGIIEKGLVTYM	3.87	0.15	0.017	0.002					3.76	0.11	12.7	2.0	7.63	0.13	63.6	1.1	> 99
915-923	IIEKGLVTY	4.06	0.13	0.0161	0.0017					2.65	0.09	11.5	2.0	6.71	0.12	83.8	1.5	> 99
918-923	KGLVTY	2.59	0.06	0.024	0.002					2.97	0.05	15.7	1.9	5.55	0.05	111.1	0.9	> 99
923-927	YMALG	2.24	0.11	0.049	0.006	1.73	0.09	8.0	1.4					3.96	0.06	99.1	1.5	> 99
923-928	YMALGL	3.19	0.18	0.038	0.006	1.76	0.15	6.7	1.8					4.94	0.10	99	2	> 99
923-930	YMALGLAL	3.0	0.2	0.022	0.006	1.85	0.19	6	2					4.80	0.18	69	3	> 99
924-929	MALGLA	3.12	0.20	0.037	0.005	0.86	0.20	2.3	1.3					3.98	0.06	79.6	1.2	> 99
924-930	MALGLAL	2.07	0.09	0.0145	0.0019	1.84	0.10	0.81	0.08					3.91	0.06	65.2	1.1	> 99
925-930	ALGLAL	1.51	0.11	0.012	0.002	1.22	0.10	0.90	0.15					2.72	0.08	54.4	1.7	> 99
928-934	LALRYVL	HDX n.d.														0		
929-934	ALRYVL	HDX n.d.														0		
929-936	ALRYVLEA	HDX n.d.														0		
931-936	RYVLEA	HDX n.d.														0		
935-945	EALRKPFGSKM	1.01	0.13	0.026	0.012	5.25	0.11	9.0	0.7					6.26	0.10	69.5	1.1	> 99
936-945	ALRKPFGSKM	1.01	0.13	0.021	0.010	5.37	0.11	8.7	0.6					6.38	0.11	79.8	1.4	> 99
937-947	LRKPFGSKMYY	0.92	0.08	0.027	0.008	5.02	0.07	8.7	0.4					5.94	0.05	65.9	0.6	> 99
946-951	YYFGIA	HDX n.d.														0		
946-952	YYFGIAA	HDX n.d.														0		
946-953	YYFGIAAL	HDX n.d.														0		
948-952	FGIAA	HDX n.d.														0		
948-953	FGIAAL	HDX n.d.														0		
952-966	ALDRFKNRLKDYPQY	3.75	0.10	0.0073	0.0005					3.62	0.04	15.9	1.5	7.38	0.10	56.7	0.8	> 99
953-966	LDRFKNRLKDYPQY	3.90	0.20	0.0055	0.0006					3.54	0.06	14.6	1.7	7.4	0.2	62.0	1.7	> 99
953-972	LDRFKNRLKDYPQYCQHLAS	8.8	0.4	0.0115	0.0014					4.7	0.2	12	3	13.5	0.4	75	2	> 99
954-966	DRFKNRLKDYPQY	3.61	0.17	0.0076	0.0008					3.42	0.07	14.2	2.0	7.03	0.17	63.9	1.5	> 99
954-972	DRFKNRLKDYPQYCQHLAS	9.1	0.4	0.0121	0.0016	5.3	0.3	8.8	1.7					14.4	0.4	85	2	> 99
973-985	ISHFMQFPHHLQE	6.5	0.6	0.036	0.007	4.1	0.6	2.8	1.1					10.6	0.2	96	2	> 99
974-985	SHFMQFPHHLQE	7.4	0.5	0.1[a]						1.9	0.4	30[a]		9.3	0.3	93	3	76

		Best-fit values																
Res. no.	Sequence	ND_1 [Da]	δND_1	k_1 [min^{-1}]	δk_1	ND_2 [Da]	δND_2	k_2 [min^{-1}]	δk_2	ND_3 [Da]	δND_3	k_3 [min^{-1}]	δk_3	ND [Da]	δND	$D_{\%}$	$\delta D_{\%}$	% AIC
977-986	MQFPHHLQEY	10	12	0.0010	0.0015	3.68	0.17	0.29[b]	0.05					13	12	170	150	> 99
978-985	QFPHHLQE	4.3	0.3	0.057	0.012					0.9	0.2	30[a]		5.2	0.2	87	3	82
979-985	FPHHLQE	3.5	0.3	0.061	0.015					1.1	0.2	30[a]		4.60	0.19	92	4	82
986-992	YIEYGQQ	5.70	0.11	0.064	0.005									5.70	0.11	94.9	1.9	64
986-999	YIEYGQQSRDPPVK	4.7	0.2	0.061	0.007	6.16	0.19	4.6	0.4					10.85	0.11	98.6	1.0	> 99
987-999	IEYGQQSRDPPVK	4.8	0.4	0.062	0.011	6.5	0.3	4.7	0.6					11.36	0.19	113.6	1.9	> 99
988-999	EYGQQSRDPPVK	3.7	0.3	0.056	0.010	5.2	0.2	4.4	0.6					8.92	0.13	99.2	1.5	> 99
989-999	YGQQSRDPPVK	2.2	0.2	0.1[a]		7.6	0.2	4.1	0.3					9.76	0.10	122.1	1.3	77

[a] Fixed values, according to [17], [96], [99], [100].

A)

MGSSHHHHHHSSGLVPRGSHMASMSDSEVNQEAKPEVKPEVKPETHINLKVSDGSSEIFFKIKKTTPL

RRLMEAFAKRQGKEMDSLRFLYDGIRIQADQTPEDLDMEDNDIIEAHREQIGGSEFNNDPFVQRKLGT

SGLNQPTFQQTDLSQVWPEANQHFSKEIDDEANSYFQRIYNHPPHPTMSVDEVLEMLQRFKDSTIKRE

REVFNCMLRNLFEEYRFFPQYPDKELHITACLFGGIIEKGLVTYMALGLALRYVLEALRKPFGSKMYYFGI

AALDRFKNRLKDYPQYCQHLASISHFMQFPHHLQEYIEYGQQSRDPPVK

B)

SEFNNDPFVQRKLGTSGLNQPTFQQTDLSQVWPEANQHFSKEIDDEANSYFQRIYNHPPHPTMSVDEV

LEMLQRFKDSTIKREREVFNCMLRNLFEEYRFFPQYPDKELHITACLFGGIIEKGLVTYMALGLALRY

VLEALRKPFGSKMYYFGIAALDRFKNRLKDYPQYCQHLASISHFMQFPHHLQEYIEYGQQSRDPPVK

Figure S1. (**A**) Sequence of the CNOT1(800-999) (dark pink) construct including the N-terminal His_6-tag, the fusion SUMO protein (cyan), and a linker (SEF). (**B**) Sequence of the final CNOT1(800-999) protein used in the studies. The residues E893 and Y900 mutated into AA or QH are underlined.

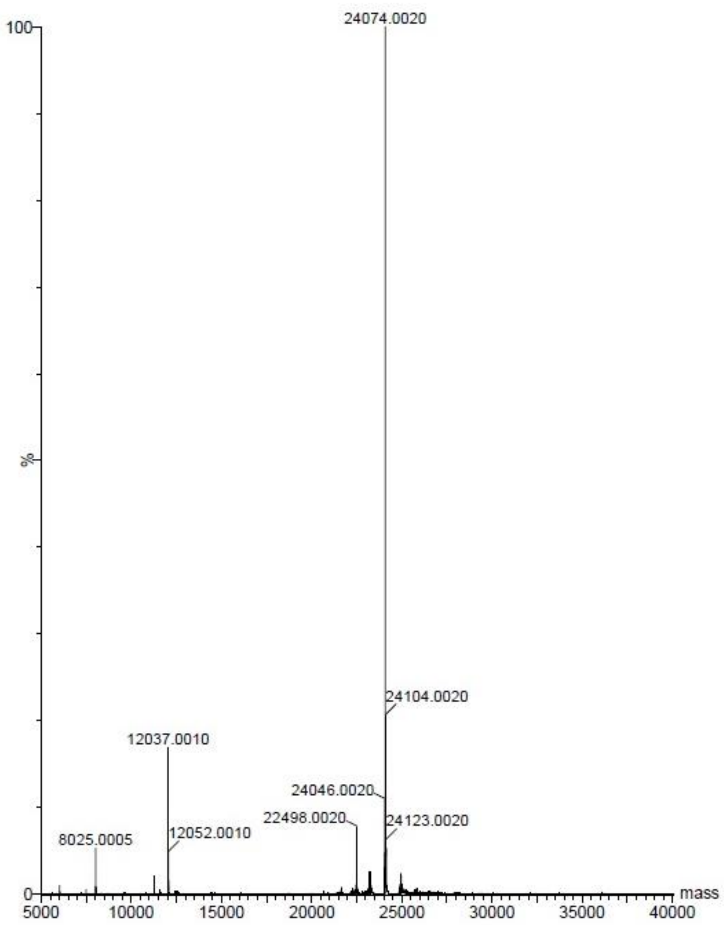

Figure S2. Deconvoluted mass spectrum of the final CNOT1(800-999) protein fragment; the sequence is shown in Figure S1 B; the predicted mass is 24074.4 Da.

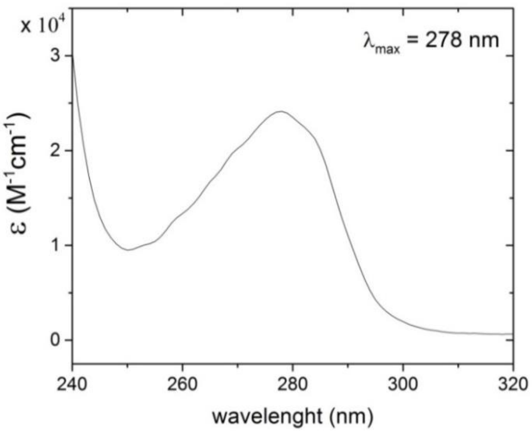

Figure S3. Absorption spectrum of wild type CNOT1(800-999) in 50 mM Tris/HCl, 150 mM NaCl, 2 mM DTT, 1 mM EDTA, pH 7.0.
